# Causes of variations of trace and rare earth elements concentration in lakes bottom sediments in the Bory Tucholskie National Park, Poland

**DOI:** 10.1038/s41598-020-80137-z

**Published:** 2021-01-08

**Authors:** Mariusz Sojka, Adam Choiński, Mariusz Ptak, Marcin Siepak

**Affiliations:** 1 Faculty of Environmental Engineering and Mechanical Engineering, Poznań University of Life Sciences, Piątkowska 94E, 60-649 Poznań, Poland; 2 Faculty of Geographical and Geological Sciences, Adam Mickiewicz University, Krygowskiego 10, 61-680 Poznań, Poland

**Keywords:** Environmental sciences, Limnology

## Abstract

The objective of this study was to analyse spatial variability of the trace elements (TEs) and rare earth elements (REEs) concentration in lake bottom sediments in Bory Tucholskie National Park (BTNP); Poland. The following research questions were posed: which factors have a fundamental impact on the concentration and spatial variability of elements in bottom sediments, which of the elements can be considered as indicators of natural processes and which are related to anthropogenic sources. The research material was sediments samples collected from 19 lakes. The concentrations of 24 TEs and 14 REEs were determined. The analyses were carried out using the inductively coupled plasma mass spectrometry (ICP-QQQ). Cluster analysis and principal component analysis were used to determine the spatial variability of the TEs and REEs concentrations, indicate the elements that are the indicators of natural processes and identify potential anthropogenic sources of pollution. The geochemical background value (GBV) calculations were made using 13 different statistical methods. However, the contamination of bottom sediments was evaluated by means of the index of geo-accumulation, the enrichment factor, the pollution load index, and the metal pollution index. The BTNP area is unique because of its isolation from the inflow of pollutants from anthropogenic sources and a very stable land use structure over the last 200 years. This study shows high variability of TE and REE concentrations in lake sediments. The values of geochemical indices suggest low pollution of lakes bottom sediments. It was found that TEs originated mainly from geogenic sources. However, the concentrations of Li, Ni, Sc, Se, Be, Se, Ag, Re, Tl, Cd, Sb and U may be related to the impact of point sources found mainly in the Ostrowite Lake. Almost all REEs concentrations were strongly correlated and their presence was linked to with geochemical processes. The elements allowing to identify natural processes and anthropogenic pollution sources were Cr, Co, Cu, Ag, Cd, Zn, Bi, Re, Ba, Al and Rb in TEs group and Nd, Gd, Yb, Lu, Eu, Dy and Ce in REEs group. The analysis shows high spatial variability of TE and REE concentrations in lake sediments. The values of geochemical indices point to low pollution of lakes sediments. The anthropogenic sources only for two lakes had an impact on concentrations of selected TEs and REEs. The analyses allowed to identify elements among TEs and REEs documenting geochemical processes and those indicating anthropogenic sources of pollution.

## Introduction

Limnological research proves that natural processes influencing the process of lake disappearance have been disturbed as a result of human activity and climate change. The transformation of land use and land cover structure contributed to the acceleration of the circulation of matter and energy in these ecosystems, including an increase in the deposition of sediments in lakes. In recent years, the potential impact of climate change, including the occurrence of extreme precipitation, has been highlighted, which can increase erosion and therefore the supply of sediments and pollutants to lakes. With sediment, various elements are deposited in the lakes which come from both natural processes and anthropogenic sources^1,2^. The comprehensive geochemical surveys can provide important information for understanding modern sedimentary processes and source-catchment interactions in any given geological setting and how they control elemental distribution in lake sediments^3^. The main factors which controls the concentration and distribution of elements in lake sediments are the catchment lithology, the degree of parent material weathering, the ratio of organic and detrital sediments, the origin of organic and inorganic components, sedimentation, sorting, post-deposition/diagenetic processes, the proportion of atmospheric deposit, moreover lake bottom morphology and depth^4,5^. The geochemical characteristics of the lake sediments allow determine a number of factors and processes responsible for elements occurrence and distribution^4^. Inorganic compounds are a kind of archive of anthropogenic activities over the years^6^. Trace elements (TEs) are indicators that document the influence of anthropogenic activity on lakes in a very good way. Their use as an indicator of long-term impacts is justified due to the very long period of their presence in the environment and their relatively stable concentration. TEs are a specific type of pollution because they are not biodegradable and accumulate in different parts of aquatic ecosystems^7^. In the natural environment the concentrations of TEs in the bottom sediments are at a level similar to the one recorded in the parental material. On the other hand, the increase in the concentration of TEs above the values recorded in parent materials and change of ratios between individual TEs may indicate their origin from anthropogenic sources. The research carried out most often includes analyses of surface and cores of lake bottom sediments^8^ and in the rivers that flow into them^9^. Measurements of TE concentrations together with dating of individual layers of sediment samples allow one to trace the history of pollution^10,11^. The assessment of the contamination of bottom sediments with TEs is performed most often in relation to the geochemical background value (GBV)^12^. Gałuszka^13^ suggested that the GBV enables one to differentiate the natural quantity of given elements in the environment from the quantity originating from anthropogenic sources. The knowledge of the GBV is essential for: defining pollution, identifying the source of contamination and for establishing reliable environmental quality criteria for bottom sediments and surface waters^14,15^. Determination of GBV has been an important issue^16,17^. However, development of GBV is not straightforward as there is no single standard or recommended methodology^18^. Kicińska and Turek^19^ stated that three group of methods are widely used for establishing the GBV: direct, indirect and integrated methods. Direct methods are based on samples collected from unpolluted areas free from anthropogenic pressure. The GBV is calculated as the arithmetic mean value, the geometric mean value or the median for the data set. In indirect methods the GBV is calculated using statistical methods. The application of indirect methods requires a large number of samples representing a homogeneous environment, characterised by similar climatic and lithological conditions. The statistical methods are based mainly on the assumption that the natural concentration of elements in the environment follows the normal or the log-normal distribution. The GBV is calculated based on the arithmetic mean (*Me*) and standard deviation (*SD*). Reimann et al.^20^ suggest that this approach (assumption of normal distribution) is not always appropriate. For this reason, to calculate the GBV value they recommend using the median value (*Md*) and the median absolute deviation (*MAD*). In addition, the box plot method is used for calculations, which enables one to eliminate outliers from the data set. Among the statistical methods, mMAD (median ± 2 Median Absolute Deviation) and TIF (Tukey inner fence) with log-transformed data are most recommended^21,22^. Moreover, graphical methods are commonly used to calculate GBV. The most commonly used worldwide are the relative cumulative distribution function method and regression analysis method^16,23^. Sahoo et al.^18^ suggested that the cumulative frequency distribution plots can be used as an alternative method to define GBV by analysing points of inflection in the curve, but it is more subjective than the mMAD and TIF methods. The integrated method is a combination of direct and indirect methods. A critical review of the methods used to determine GBV was presented in their paper^24^. The GBV values are used to calculate geochemical indices, which allow one to answer a series of questions: What is the pollution level of bottom sediments? What are their potential eco-toxicological effects? What was or is the human pressure on the lake ecosystem^25,26^? There are many different approaches to evaluate the pollution status of TEs in sediments, such as: sediment quality guidelines (SQG), geo-accumulation index (I_geo_), enrichment factor (EF), pollution load index (PLI), sediment pollution index (SPI) and sediment enrichment factor (SEF)^23^. Somewhat more problems are related to the analysis of TE sources in bottom sediments. For this purpose, research is carried out in order to identify sources of pollution. There is often an overlap between different sources of pollution^27^. Therefore, various statistical analyses are carried out to highlight the share of particular sources of pollution in the total transport of TEs to lakes^28–31^. Geochemical knowledge on the distribution and migration of elements is crucial to identify their origin^14^. Nevertheless, it is important to choose the more suitable method to determine GBV for avoiding any unrealistic assessment which may have serious consequences^5^.

In recent years, the use of new advanced technologies has resulted in a strong interest in rare earth elements (REEs)^32^. The main sources of REE include medical facilities, petroleum refining, mining and technology industries, fertilizers, livestock feeds, and electronic wastes and recycling plants^33^. Based on concentrations of REEs in bottom sediments geochemical parameters are calculated. They provide information about the provenance and moreover reflect the fractionation degree in the geochemical processes^8^. Moreover, the REE concentrations in sediments can be used to identify potential pollution sources^34^. Gwenzi et al.^33^ suggested that there is a lack of comprehensive reviews on the anthropogenic sources, environmental behaviour, and public and ecological health risks of REEs.

The relationship between natural and anthropogenic altered concentrations of chemical species involves many factors in the geosciences, environmental and biological sciences, toxicology, and other related disciplines^13^. The analysis of the literature on anthropogenic pressure on lakes shows that there are relatively few so-called anthropogenic-free lakes around the world. Lakes located in national parks could possibly be considered a small group of these. However, they are also supplied by rivers which are exposed to inflows of pollutants from the catchment area^35–38^. These are pollutants that are supplied transgenically and their reduction involves taking action to prevent them over very large areas. In Poland there are 23 national parks with 95 lakes^39^. Poor water status in lakes located in national parks results from their location within the range of intensive human pressure zone. On the other hand, the energy sector in Poland is largely based on the use of fossil fuels, i.e. black and brown coal. This may be associated with a high supply of pollutants to the atmosphere, which may subsequently be deposited in surface waters in the form of dry or wet deposits.

Lakes located in the BTNP are specific in this context. The BTNP is located in the central part of the Brda River within the area drained by the Struga Siedmiu Jezior River. The catchment area of the Struga Siedmiu Jezior covers 85.5% of the area of the BTNP and can therefore be considered unique. This is due to the total limitation of the inflow of pollutants to the lakes from anthropogenic sources via roads and a very stable land use structure (mainly forest) over the last 200 years^40^.

The aims of the study were to: (1) analyse TE and REE concentrations in bottom sediments of BTNP lakes, (2) determine GBV values for TEs and REEs, (3) assess bottom sediment pollution by TEs and REEs, (4) analyse the sources of individual elements in bottom sediments, (5) identify elements that may be indicators of natural and anthropogenic processes. Chemical analyses of bottom sediments in the area of the BTNP in such a wide range of lakes and parameters (TEs and REEs) are performed for the first time, and therefore this study may be a reference point for future geochemical surveys.

## Material and methods

### Study site

**Figure 1:**
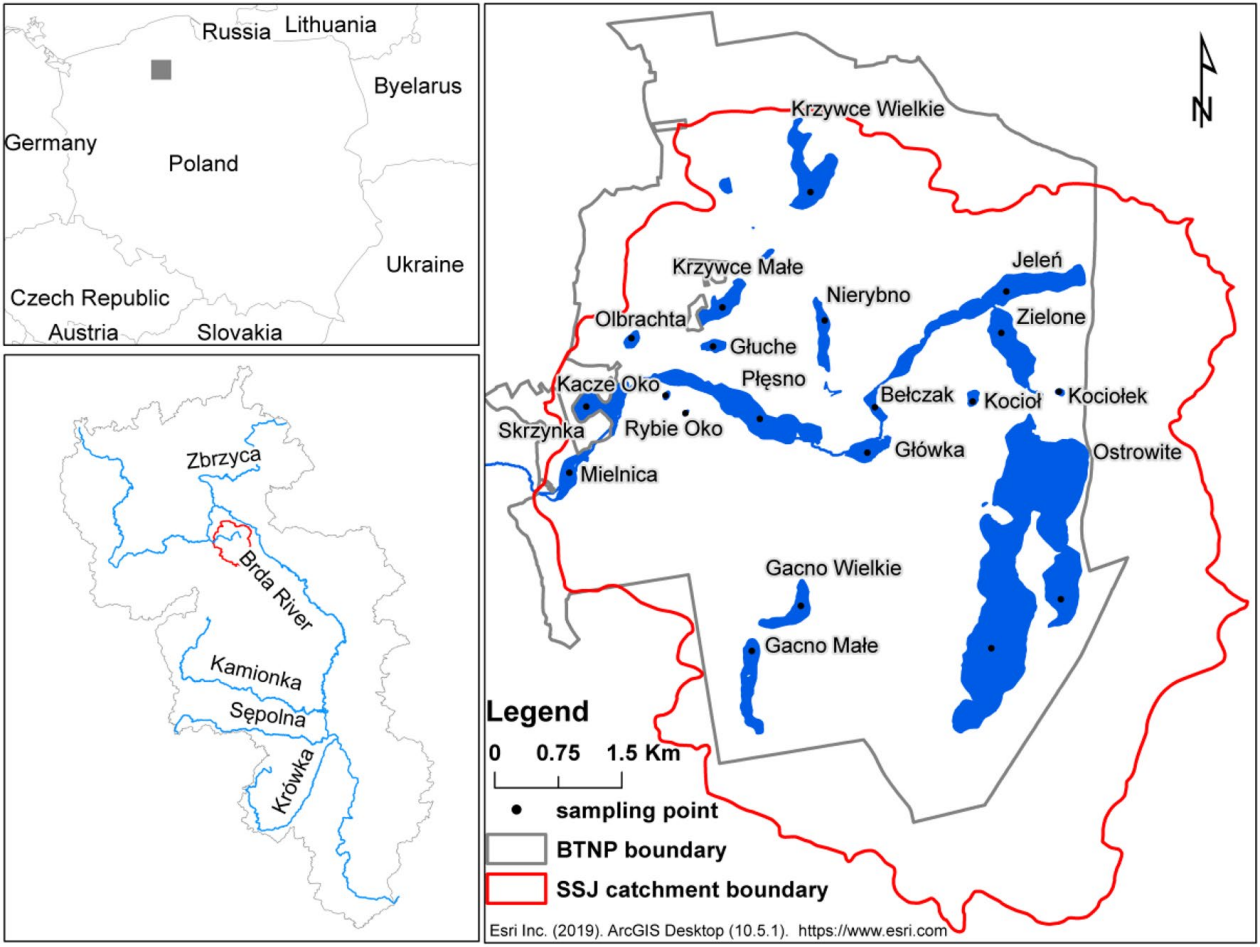
Study site location. Figure generated in ArcMap 10.5 (https://desktop.arcgis.com/en/arcmap/).

The Bory Tucholskie National Park (BTNP) was established in 1996. The BTNP is located in the central part of the Brda catchment area within the Struga Siedmiu Jezior catchment. The Struga Siedmiu Jezior stream flows into Charzykowskie Lake, which is located on the Brda River, the left tributary of the Vistula River, the largest river in Poland. The area of the BTNP is 46.13 km^2^. The most valuable parts of the BTNP are 24 lakes with a total area of 5.37 km^2^. The Ostrowite, Zielone, Jeleń, Bełczak, Główka, Płęsno, Skrzynka and Mielnica Lakes are located in the Struga Siedmiu Jezior stream (Fig. 1). The lakes Krzywce Wielkie, Błotko, Głuche and Krzywce Małe are connected by an artificial channel which periodically leads the water to the lake Skrzynka. Other lakes do not have direct contact with rivers. The total length of the BTNP watercourses is 12.4 km, which gives in relation to the catchment area a river network density of 0.27 km km^−2^.

The study included 19 lakes (Table 1). Ostrowite Lake is the deepest lake in the BTNP with a maximum depth of 43 m, whereas Mielnica Lake with a maximum depth of 1.2 m is the shallowest. The lakes’ surface area varies from 0.3 to 280.7 × 10^4^ m^2^ and volume from 5.5 × 10^3^ to 29,989.8 × 10^3^ m^3^. The ratio of the total area of the lake drainage basin and the area of the lake is from 6.2 to 544.2 for Ostrowite and Bełczak lakes, respectively. The lake coefficient indicates the potential amount of matter supply from the catchment area (Table 1). The BTNP includes oligotrophic, mesotrophic, eutrophic and dystrophic lakes^41,42^. According to the hydrological typology, they are flow-through, exorheic and endorheic lakes, and according to the thermal typology, the lakes are stratified, partially stratified and not stratified. The unique feature of the BTNP is the occurrence of five lobelian lakes—Krzywce Wielkie, Gacno Małe, Gacno Wielkie, Nierybno and Głuche. The name “*lobelian lakes*” comes from the Latin name of the taxon—*Lobelia dortmanna L.* This species is very rare and hence has been included in the Polish Red Data Book of Plants.

**Table 1:** Basic parameters of lakes in the BTNP.

Lake	Area (Area) (ha)	Volume (Vol) (10^3^ m^3^)	Average depth (AD) (m)	Maximum depth (MD) (m)	Catchment area (km^2^)^a^	Lake coefficient (LC) (–)	Lithology (% contribution in catchment area/formations)	Total organic matter (TOM) (%)	Bottom sediments sample number	Location of sampling sites
Ostrowite	280.7	29,989.8	10.7	43	17.3	6.2	69/A, 7/B, 11/E, 6/C, 10/G	34.9	1^c^	53° 47′, 17° 35′
								29.4	2^b^	53° 47′, 17° 36′
Jeleń	48.8	2067.3	4.2	10.7	23	47.1	95/A, 5/B	41.3	3	53° 49′, 17° 35′
Płęsno	47.8	2254.1	4.7	11	24.9	52.1	100/A	24.1	4	53° 48′, 17° 33′
Krzywce Wielkie	26.5	1724.1	6.5	15	2.4	9.1	94/A, 5/B, 1/C	52.9	5	53° 50′, 17° 33′
Zielone	25.5	2253.4	9	20.5	18.6	72.9	85/A, 15/B	36.5	6	53° 49′, 17° 35′
Skrzynka	21.1	369.8	1.8	4.6	37.9	179.6	75/A, 25/B	28.0	7	53° 48′, 17° 31′
Gacno Małe	15.5	481.2	3.1	5.6	1.8	11.6	85/A, 15/B	63.5	8	53° 47′, 17° 33′
Gacno Wielkie	13.5	376	2.7	6.1	5.8	43.0	75/A, 25/B	62.3	9	53° 47′, 17° 34′
Krzywce Małe	11.8	621.7	5.3	10	6.1	51.7	82/A, 3/B, 15/C	44.7	10	53° 49′, 17° 32′
Mielnica	11.3	82.7	0.7	1.2	41.0	362.8	80/A, 18/B, 2/E	29.8	11	53° 48′, 17° 31′
Nierybno	10.1	260.0	2.5	6.3	1.4	13.9	88/A, 2/B, 10/D	66.9	12	53° 49′, 17° 33′
Główka	8.0	299.5	3.7	11	24.8	310.0	67/A, 8/B, 25/D	29.7	13	53° 48′, 17° 34′
Bełczak	4.3	147.2	3.4	6	23.4	544.2	70/A, 30/D	39.7	14	53° 48′, 17° 35′
Głuche	3.3	104.5	3.2	6.5	0.5	15.2	80/A, 20/B	29.3	15	53° 49′, 17° 32′
Olbrachta	2.6	67.4	2.6	3.7	6.5	250.0	60/A, 40/C	55.1	16	53° 49′, 17° 31′
Kocioł	2.5	170.7	6.8	11	0.3	12.0	100/A	67.2	17	53° 48′, 17° 36′
Kociołek	0.7	16	2.2	5.3	0.1	14.3	100/A	72.9	18	53° 49′, 17° 36′
Kacze Oko	0.5	5.5	0.9	1.3	0.1	20.0	55/A, 45/B	64.3	19	53° 48′, 17° 32′
Rybie Oko	0.3	11.2	3.5	6.1	0.2	66.7	65/A, 35/B	60.1	20	53° 48′, 17° 33′

*A* outwash plain sands and gravels; *B* aeolian sands; *C* peats; *D* aggradate muds; *E* silts, limnic sands and chalks; *F* fluvial sands and gravels; *G* boulder loams.

^a^Woronko and Nowicka^43^.

^b^Eastern part.

^c^Western part.

The relief was formed during the period of the Baltic glaciation. The morphology is variable due to development of tunnel valleys, kettle holes and aeolian dunes^44^. The lowest point is located near Lake Mielnica (119.46 m a.s.l.), and the highest is within the range of hills located in the southern part of the catchment (148.75 m a.s.l.). The highest slopes occur in the hillside area of tunnel valleys and kettle holes. Sandy glacial sediments are the main parent material of the soils. The largest area is occupied by sands (90.2%), followed by loamy and loamy sands (7.0%). In addition, hydrogenic soils (2.8%) represented by mudslides and peats occur within the lake valley area. The lithological structure of the direct lake catchment areas is presented in detail in Table 1. The outwash plain sands and gravels or aeolian sands generally dominate in the direct lake catchment. On the other hand, in Krzywce Wielkie and Olbrachta lakes, peats have a high proportion, and in the catchment areas of Główka, Bełczak and Nierybnozna lakes, there are aggradate muds. One of the unique characteristics of the BTNP is its location, which corresponds 85.5% to the catchment area of the Struga Siedmiu Jezior stream. The lack of transitional rivers makes the lakes isolated from point and non-pollution sources. The atmospheric precipitation and dry deposition may be the only sources of pollution. The BTNP area is dominated by forest areas (86.8%) and water bodies (11.2%). A small part of the catchment is occupied by arable land and built-up areas (total 0.1%) and meadows and pastures (1.9%). The forests area in the BTNP has increased from 75.2 to 86.8% over the last 120 years, mainly due to the reduction of arable land from 9.8% to less than 0.1%. Arable land, meadows and a built-up area are located in the vicinity of the western part of Ostrowite Lake. In addition, there is a road network near the lakes which may be a potential source of pollution. The length of roads in the BTNP area is 116 km, which gives a density of 2.53 km km^−2^.

### Sample collection and preparation

Samples of bottom sediments for chemical analyses were collected on 7 and 8 August 2017 from 19 lakes located in the BTNP area (Fig. 1). Samples were taken at the deepest point of each lake and in the case of Ostrowite Lake two samples were taken at the deepest points of the two western and eastern parts. Bottom sediment samples of 10 cm in depth were collected by a Nurek-1 sampler (Mera-Błonie, Gdańsk, Poland) into polyethylene (PE) containers. The sample numbers are presented in Table 1. The samples after transport to the laboratory were divided into two parts. The first part of the sediment was dried at a temperature of 21 °C. Subsequently, the total organic matter (TOM) content was determined. The TOM was measured by the Tiurin method with wet oxidation, followed by ferrous ammonium sulphate titration^45^.

### Chemical analysis

The second part of the sediment samples were dried at 105 °C in a Binder FD 53 (Binder GmbH, Germany). Dry samples were mineralized with aqua regia prepared from HNO_3_:HCl (1:3 v/v) by Merck (Merck, Darmstadt, Germany). The samples were extracted using the MARS 5 Xpress (CEM, Matthews, NC, USA) microwave digestion system. The concentrations of TEs (Li, Be, Al, Sc, V, Cr, Co, Ni, Cu, Zn, As, Se, Rb, Mo, Ag, Cd, Sb, Ba, Pb, Bi, Th and U) and REEs (La, Ce, Pr, Nd, Sm, Eu, Gd, Tb, Dy, Ho, Er, Tm, Yb and Lu) in the bottom sediment samples with a fraction of < 2 mm were determined by inductively coupled plasma mass spectrometry (ICP-QQQ), model 8800 Triple Quad (Agilent Technologies, Japan). This instrument is equipped with a MicroMist nebulizer and a Peltier-cooled (2 °C) Scott-type spray chamber for sample introduction. This instrument contains an octopole-based collision/reaction cell, located in between two quadrupole analyzers. It was operated in a gas mode, with O_2_ flowing 0.3 mL/min (30%) and He flowing at 5 mL/min. The analytical procedure is described in previous papers^2,29,31^. The reagents used were analytically pure, and the water was purified to the resistivity of 18.2 MΩ^.^cm (at 25 °C) in a Direct-Q UV3 Ultrapure Water Systems apparatus (Millipore, France). During the determinations by the ICP-QQQ techniques, standard solutions produced by VHG Labs, Inc. (Manchester, England) were used. We measured the Certified Reference Material CRM no. LGC 6187 used for River sediments (Manchester, England), and high compliance with reference values was found (see Supplementary Table S1 online). MassHunter software for ICP-QQQ (Agilent Technologies, Japan) was used to control the instrument and to process the data^29^.

### TE and REE concentrations in bottom sediments

In the first stage the general characteristics of TE and REE concentrations were determined. For this purpose, the values of minimum, average, maximum concentration and standard deviation were calculated. Additionally, the percentile values were calculated for all elements (1%, 5%, 10%, 25%, 50%, 75%, 90%, 95% and 99%). Then TEs and REEs were analyzed for their variability, type of distribution and occurrence of outlier observations. The Grubbs-Beck test (G-B) was used to detect outlier observations. The distribution of TEs and REEs data was investigated using one-sample Kolmogorov–Smirnov test (or the K–S test of normality). The G–B and K–S tests were performed at the significance level of 0.05.

### Determination of GBV—comparison of different approaches

GBV calculations were made using 13 different statistical methods (Table 2).

**Table 2:** Methods used to determine geochemical background value.

Designation	Calculation procedure	References
M2SD	*Me* + *2 SD*	^46^
LTM2SD	Log-transformed data *Me* + *2 SD* Back-transform the resulting threshold value	This study
ORM2SD	Removing outliers from data (Grubbs–Beck test) *Me* + *2 SD*	This study
ORLTM2SD	Removing outliers from data (Grubbs–Beck test) Log-transformed data *Me* + *2 SD* Back-transform the resulting threshold value	This study
M2MAD	*Md* + *2 MAD*	^20^
LTM2MAD	Log-transformed data *Md* + *2 MAD* Back-transform the resulting threshold value	^21,22^
TIF	*Q*_*3*_ + *1.5 IQR* where Q_3_ (3rd quartile equivalent to the 75th percentile), and IQR is the interquartile range (75th–25th percentile)	^47^
LTTIF	Log-transformed data *Q*_*3*_ + *1.5 IQR* where Q_3_ (3rd quartile equivalent to the 75th percentile), and IQR is the interquartile range (75th–25th percentile) Back-transform the resulting threshold value	^21^
90th	90th percentile	^48^
95th	95th percentile	^49^
98th	98th percentile	^50^
CDFIP	CDF inflexion points detection Inflexion points were determined by examining the CDF vs. TEs/log-TEs and log-REEs data under linear regression model for P < 0.05 and R^2^ > 0.95. The GBVs were the upper inflexion points for TEs and REEs	^51^
NLRR	Normalization based linear regression relationship between TEs or REEs and a reference element for V and La, respectively $\left( {{\text{NLRR}} = {\text{a}}\;\overline{V\;or\;La} + {\text{b}}} \right)$. The GBV for TEs and REEs was calculated on the basis of equations and *Md* + *2 MAD* value	^51,52^

The first method used is one of the most frequently described in the literature, which uses the mean value (*Me*) and standard deviation (*SD*)—M2SD (*Me* + *2SD*). With respect to this method there are also many critical comments, which result from the assumption of a priori normality of the distribution of concentrations of analysed elements. In this study it was found that some of the TEs were normally (Al, V, Cr, Co, As, Ba, Re, Pb, Bi and Th) or log-normally distributed (Li, Be, Sc, Ni, Cu, Zn, Se, Rb, Mo, Ag, Cd, Sb and U). However, the REEs were log-normally distributed. For this reason, an attempt was made to modify the previously used method. For this reason the data were therefore log-transformed (log10(x)). On the basis of log-transformed data, the values of the *Me* + *2SD* were calculated. Then the results were back-transformed—LTM2SD. Taking into account the other critical comments concerning the calculation of GBV by the M2SD method, it was decided to remove outliers identified by the Grubbs–Beck test. Then, on the basis of data excluding outliers, GBV calculations were made using a standard procedure based on *Me* and *SD* (ORM2SD) and in combination with logarithmic transformation (ORLTM2SD). Another way that is recommended when calculating GBV because of the occurrence of distributions other than normal and outlier observations is to use formulas based on the median value (*Md*). Therefore, the M2MAD method based on the median value (Md) and median absolute deviation (*MAD*) was used to determine GBV. Reimann et al.^21^ and Salomão et al.^22^ recommended applying LTM2MAD method based on the median value (Md) and median absolute deviation (MAD) with log-transformed dataset. In addition, the Tukey Boxplot method (Tukey inner fence, TIF) was used, which is based on the following values: 75th percentile (Q_3_) and inter quartile range (IQR). The IQR is calculated as the difference between the 75th percentile and 25th percentile (*Q*_3_–*Q*_1_) multiplied by the value 1.5^47^. Reimann et al.^21^ recommended also to use LNTIF method, which is analogous to the TIF method, however, a log-transformed dataset are used. Moreover, for each TE and REE a cumulative distribution function (CDF) was developed, from which the values of the 90th, 95th and 98th percentile were obtained. These values were considered to be GBVs. The CDF was also used to find inflexion points. The lower inflexion point represents the upper limit of natural origin concentrations, and the higher inflexion point represents the lower limit of abnormal concentrations^51^. The inflexion points in a CDF were determined by examining the CDF vs. TEs and/log-TEs and log-REEs data under the linear regression model for P < 0.05 and R^2^ > 0.95. The data of Li, Be, Sc, Ni, Cu, Zn, Se, Rb, Mo, Ag, Cd, Sb and U and REEs were therefore log-transformed prior to the CDF plotting. The TEs and REEs concentrations were plotted on the x-axis and the cumulative frequency was plotted on the y-axis. The maximum value was removed to obtain linearity of the data; this procedure was repeated until the above two criteria were met, and the maximum value within the regression line will represent the inflexion^51^. Finally the first point deviating from the regression line was chosen as the upper inflexion point, which was considered to be the baseline value for the selected TEs and REEs. In the last NLRR method was carried out to find the linear relationship between the contents of each TE and REE elements and reference elements^53,54^. Reference elements proposed by Wei and Wen^51^ included Al, Fe, La, Li, Sc, Ti and V. In order to select the best reference elements, correlation analysis was carried out, and showed that for TEs the reference element is V and for REEs the reference element is La. The choice of these elements resulted from the degree of correlation between the individual TEs or REEs and the reference elements. The linear relationships between each TE and V and REE and La were determined, in the form of y = ax + b^55^. As proposed by Covelli and Fontolan^53^ the data beyond 95% confidential intervals of the linear regression were removed as outliers. Thus, the developed linear regressions were used to calculate the GBV of TEs and REEs based on V and La concentrations respectively.

The calculated GBV values were compared with those provided by Salminen et al.^56^ in the European Geochemical Atlas. GBV values were obtained for the location corresponding to the BTNP.

### Assessment of bottom sediment contamination

The assessment of bottom sediment pollution by TEs and REEs was performed on the basis of four commonly used indices: the index of geo-accumulation (I_geo_), the enrichment factor (EF), the pollution load index (PLI), and the metal pollution index (MPI). I_geo_ and EF are single indices, while PLI and MPI are integrated indices. For I_geo_, EF and PLI the concentrations of TEs and REEs in bottom sediments are related to the GBV. Formulas for calculating I_geo_, EF and PLI are widely used and can be found in the publications^57–59^. The limit values for the assessment of bottom sediment contamination have been assumed to be Martin and Meybeck^58^, Harikumar et al.^60^ and El-Sayed et al.^61^.

The only index that does not use GBV is MPI. The MPI was used to compare the total TE and REE contents between different lakes. The MPI was calculated as follows:

$${\text{MPI}} = \left( {{\text{C}}_{{{\text{n}}1}} \cdot {\text{C}}_{{{\text{n}}2}} \cdot \cdots \cdot {\text{C}}_{{{\text{ni}}}} } \right)^{{\frac{1}{{\text{i}}}}} ,$$

where *C*_*n*_ are concentrations of TEs and REEs in the sample and i is the number of analyzed metals^62,63^. The GBV for TEs and REEs was assumed on the basis of our own research results. CDFIP methodology based on CDF and inflexion point was assumed as a reference. The calculated GBVs for lake bottom sediments in BTNP are presented in Table 4. In order to present the results of the I_geo_, EF, PLI and MPI indices, a Python language program (GeoVIS) was developed, which allows the automatic creation of heat maps.

### Calculation of REE anomalies

The analysis of REE concentrations in bottom sediments started with the development of standardized graphs with respect to concentrations in chondrite^64^. The Eu, Gd and Yb anomalies were preliminarily identified on standardised graphs against REE concentrations in chondrite. Then the analysis of the REE concentration anomalies was performed. Anomalies in the REE concentration enable one to identify potential sources of pollution and the influence of natural processes on their concentration^32^. In order to calculate the Eu, Gd and Yb anomaly, the equations proposed by Benabdelkader et al.^65^ were used. In the last stage the REE values were calculated for three groups: light (LREE—sum of La, Ce, Pr and Nd concentrations), medium (MREE—sum of Sm, Eu and Gd concentrations) and heavy (HREE—sum of Tb, Dy, Ho, Er, Tm, Yb and Lu concentrations).

### Statistical analysis

The collected data were subjected to multivariate statistical analyses. The multivariate analysis was carried out on data that were transformed using centred log-ratio (clr). The clr transformation destroys the closure effect and ‘opens’ or ‘un-constrains’ the data to reveal inherent patterns in the data structure that can provide straight forward interpretation^66–68^.

Cluster analysis (CA) was conducted to divide the lakes to the groups characterized by the highest similarity in terms of TE and REE concentrations in bottom sediments. CA analysis was performed using the Ward method and the Euclidean distance square as a measure of similarity. A dendrogram was used to visualize the results of the CA analysis. The results of clustering lakes on the basis of TE and REE concentrations in lake bottom sediments was presented using the standardized linkage distance (D_link_/D_max_). The mean and median values were calculated for the groups and subgroups. To assess the statistical differences among groups of lakes taking into account concentrations of TEs and REEs in bottom sediments a non-parametric test of the variance (Kruskal–Wallis, K–W) and Dunn’s tests as post hoc procedures (P ≤ 0.05) were performed.

In order to select the next method of statistical analysis, detrended component analysis (DCA) was carried out. DCA was conducted to assess the environmental factors’ distribution pattern. The DCA indicated that the TE and REE concentrations and environmental factors were in linear distribution. Ter Braak and Smilauer^69^ suggested that in such cases principal component analysis (PCA) is more appropriate for future analysis. The number of significant principal components (PC) was selected on the basis of a Kaiser criterion—eigenvalues higher than 1. The correlation between PC and TEs and REES was classified according^70^. The correlation coefficient values > 0.75, 0.75–0.50 and 0.50–0.30 indicate strong, moderate and weak relationships. In order to identify the elements that best allow for the assessment of the contamination of bottom sediments, the multiple regression method was applied. Input data were all TEs and REEs (independent variables) and values of aggregated indexes PLI and MPI (dependent variables). The definition of the multiple regression model was carried out using the progressive stepwise method. The elements which were included in the model and were significant at the level of 0.05 were selected as relevant for the characteristics of bottom sediment contamination.

The Grubbs–Beck test, Kruskal–Wallis (K–W) and Dunn’s tests, Kolmogorov–Smirnov test and cluster analysis (CA) were carried out using STATISTICA software version 13.1 (Dell, Inc. 2016, Dell Statistica). Canoco software version 5.0 (Microcomputer Power, Ithaca) was used for the PCA and DCA. The clr transformation was carried out using open source statistical software R version 3.6.1 (R Development Core Team, 2009).

## Results

In the first stage of the analysis, the values of minimum, mean, median, maximum and standard deviations were calculated. In addition, CDF were developed from which the values of percentiles 1, 5, 10, 25, 50, 75, 90, 95 and 99 were obtained (Table 3). Based on the mean values, the TE concentrations were ordered in the following ascending order: Tl < Se < Th < Ag < Sc < Bi < Be < Re < Sb < U < Co < Cd < Rb < Mo < Cr < As < Cu < Ni < Li < V < Pb < Ba < Z < Al. Among the analysed TEs the highest variability was observed in the concentrations of Se, Ag, Cd, Sb and U. The lowest variability was observed for Al, Ba and Th. On the other hand, the concentrations of REEs in the bottom sediments of lakes were in the following ascending order Lu < Tm < Ho < Tb < Yb < Yb < Eu < Er < Dy < Sm < Pr < Gd < La < Nd < Ce. In the case of REEs the variability was lower than in TEs. The highest variability was observed for Er, Tm and Lu and the lowest for La, Pr, Nd, Ho and Yb.

**Table 3:** TE and REE concentrations in lake bottom sediments in mg kg^−1^.

Parameter	Min	Mean	Max	SD	Percentiles									Outlier^a^	Distribution type
					1	5	10	25	50 median	75	90	95	99		
**Trace elements—TEs**															
Li	0.22	2.00	6.63	1.86	0.22	0.22	0.34	0.69	1.22	3.02	5.11	6.22	6.63	1	LN
Be	0.03	0.11	0.49	0.10	0.03	0.03	0.03	0.06	0.08	0.12	0.19	0.34	0.49	1	LN
Al	401	2543	4898	1335	401	729	1062	1270	2600	3517	4499	4715	4898		N
Sc	0.021	0.100	0.269	0.072	0.021	0.022	0.028	0.050	0.076	0.137	0.221	0.248	0.269	1	LN
V	0.79	3.61	8.34	2.16	0.79	0.99	1.20	1.90	3.10	4.77	7.07	8.32	8.34		N
Cr	0.20	1.28	2.96	0.84	0.20	0.21	0.28	0.57	1.22	1.59	2.75	2.87	2.96		N
Co	0.08	0.44	1.04	0.28	0.08	0.09	0.11	0.21	0.41	0.61	0.86	0.96	1.04		N
Ni	0.58	1.71	5.31	1.16	0.58	0.60	0.70	1.00	1.38	1.98	3.24	4.53	5.31	1, 2	LN
Cu	0.16	1.66	5.35	1.51	0.16	0.17	0.22	0.67	1.10	2.20	4.40	5.22	5.35	5, 9	LN
Zn	13.7	41.4	105.3	24.8	13.7	15.1	18.1	24.1	33.2	52.8	78.6	94.5	105.3	15	LN
As	0.15	1.34	3.73	0.89	0.15	0.23	0.33	0.67	1.32	1.68	2.50	3.35	3.73	10	N
Se	0.01	0.09	0.63	0.14	0.01	0.02	0.03	0.04	0.06	0.08	0.18	0.45	0.63	1, 2	LN
Rb	0.17	0.74	1.78	0.48	0.17	0.18	0.22	0.40	0.63	0.96	1.57	1.75	1.78		LN
Mo	0.07	1.12	3.67	0.10	0.07	0.15	0.25	0.37	0.81	1.51	2.70	3.56	3.67	6, 8	LN
Ag	0.02	0.10	0.460	0.11	0.02	0.03	0.03	0.05	0.06	0.09	0.24	0.37	0.46	1, 2, 9	LN
Cd	0.072	0.545	2.473	0.577	0.072	0.072	0.073	0.124	0.403	0.646	1.283	1.893	2.473	1	LN
Sb	0.03	0.26	1.36	0.30	0.03	0.03	0.04	0.09	0.18	0.30	0.53	0.97	1.36	1	LN
Ba	8.24	16.3	30.9	6.22	8.24	8.32	8.96	11.2	15.8	19.2	25.7	29.6	30.9		N
Re	0.011	0.192	0.463	0.132	0.011	0.017	0.023	0.091	0.181	0.289	0.380	0.430	0.463		N
Tl	0.005	0.059	0.260	0.058	0.005	0.007	0.010	0.019	0.057	0.072	0.109	0.201	0.260	1	LN
Pb	0.14	5.37	14.6	3.94	0.14	0.60	1.11	2.85	4.26	7.59	11.6	14.2	14.6		N
Bi	0.021	0.109	0.252	0.067	0.021	0.025	0.030	0.056	0.107	0.152	0.206	0.250	0.252		N
Th	0.028	0.096	0.165	0.037	0.028	0.037	0.047	0.073	0.090	0.122	0.152	0.164	0.165		N
U	0.015	0.335	1.889	0.444	0.015	0.015	0.017	0.053	0.230	0.420	0.808	1.443	1.889	1, 2	LN
**Rare earth elements—REEs**															
La	0.262	1.384	4.589	1.015	0.262	0.305	0.349	0.715	1.146	1.763	2.533	3.687	4.589	1	LN
Ce	0.553	3.146	10.90	2.473	0.553	0.651	0.761	1.516	2.520	3.952	6.191	9.043	10.90	1	LN
Pr	0.064	0.331	0.983	0.235	0.064	0.070	0.081	0.168	0.283	0.438	0.652	0.878	0.983	1	LN
Nd	0.250	1.398	4.861	1.079	0.250	0.301	0.355	0.675	1.174	1.819	2.637	3.907	4.861	1	LN
Sm	0.042	0.279	0.992	0.231	0.042	0.049	0.057	0.130	0.227	0.361	0.565	0.848	0.992	1	LN
Eu	0.009	0.061	0.213	0.052	0.009	0.010	0.013	0.021	0.049	0.078	0.133	0.194	0.213	1, 2	LN
Gd	0.075	0.404	1.770	0.381	0.075	0.090	0.109	0.181	0.316	0.476	0.766	1.350	1.770	1, 2	LN
Tb	0.008	0.042	0.192	0.041	0.008	0.009	0.011	0.019	0.033	0.049	0.079	0.143	0.192	1, 2	LN
Dy	0.019	0.202	0.850	0.189	0.019	0.027	0.036	0.078	0.182	0.249	0.391	0.665	0.850	1	LN
Ho	0.006	0.034	0.100	0.026	0.006	0.006	0.007	0.015	0.031	0.042	0.076	0.098	0.100	1, 2	LN
Er	0.006	0.103	0.529	0.116	0.006	0.010	0.016	0.038	0.088	0.118	0.195	0.391	0.529	1, 2	LN
Tm	0.001	0.011	0.053	0.012	0.000	0.001	0.002	0.004	0.010	0.014	0.022	0.039	0.053	1	LN
Yb	0.010	0.059	0.129	0.038	0.010	0.014	0.018	0.020	0.055	0.084	0.113	0.124	0.129		LN
Lu	0.001	0.011	0.058	0.013	0.001	0.001	0.001	0.004	0.008	0.013	0.021	0.043	0.058	1, 2	LN

*N* normal distribution, *LN* log-normal distribution.

^a^Sample numbers as shown in Table 1.

The Grubbs–Beck test showed that there were 21 outlier observations in the TE dataset (Table 3). Outlier values were found in the Li, Be, Sc, Ni (two values), Cu (two values), Zn, As, Se (two values), Mo (two values), Ag (three values), Cd, Sb, Tl and U (two values). Ten outlier values were recorded in samples of bottom sediments from the western part of Ostrowite Lake (sampling point no. 1—Li, Be, Sc, Ni, Se, Ag, Cd, Sb, Tl and U), four outlier values in the eastern part of Ostrowite Lake (sampling point no. 2—Ni, Se, Ag and U), two outlier values in Gacno Wielkie Lake (Cu and Ag) and one outlier value in Krzywce Wielkie Lake (Cu), Zielone Lake (Mo), Gacno Małe Lake (Mo), Krzywce Małe Lake (As) and Głuche Lake (Zn) (Table 3). REE analysis revealed 19 outliers in the data set. Among 14 REEs in 13 cases outliers were observed. Only in the case of Yb were no outliers observed. Most outlier observations were recorded in the sediment samples from the western part of Ostrowite Lake (no. 1—La, Ce, Pr, Pr, Nd, Sm, Eu, Gd, Tb, Dy, Ho, Er, Tm, Lu). Moreover, six outlier values (Eu, Gd, Tb, Ho, Er and Lu) were found in the eastern part of Ostrowite Lake (no. 2). Among the analysed TEs the following elements had normal distributions: Al, V, Cr, Co, As, Ba, Re, Pb, Bi and Th. The other TEs elements—Li, Be, Sc, Ni, Cu, Zn, Se, Rb, Mo, Ag, Cd, Sb and U—were log-normally distributed. The distributions were right-skewed; mean values (Me) were higher than median values (Md). All REEs were log-normally distributed.

Statistical analysis showed that in the TE and REE data set there are elements that are characterized by different variability and distributions. Moreover, there are also outliers among them. Taking this into account, it was decided to test different methods of GBV calculation in order to illustrate the uncertainty of such analyses. Moreover, it should be noted that the analysed lakes are located in a small area, which is separated from anthropogenic sources, including transgenic ones. The GBV results obtained by different methods are shown in Table 4. The number of values exceeding the set GBV values is given in brackets.

**Table 4:** GBV calculated by different methods.

Parameter	M2SD	LTM2SD	ORM2SD	ORLTM2SD	M2MAD	LTM2MAD	TIF	LTTIF	90th	95th	98th	CDFIP	NLRR	EGA
**Trace elements—TEs**														
Li (1)	5.73 (2)	9.1 (0)	4.87 (2)	7.93 (0)	3.47 (4)	5.19 (2)	6.06 (1)	22.25 (0)	4.53 (2)	5.86 (1)	6.32 (1)	3.36 (4)	3.66 (3)	6.08 (1)
Be (1)	0.31 (1)	0.35 (1)	0.19 (2)	0.26 (1)	0.19 (2)	0.18 (2)	0.21 (1)	0.33 (1)	0.18 (2)	0.21 (1)	0.38 (1)	0.19 (1)	0.24 (1)	0.78 (0)
Al^a^ (0)	5214 (0)	7608 (0)	5214 (0)	7862 (0)	5966 (0)	6517 (0)	6798 (0)	15,274 (0)	4472 (2)	4551 (1)	4759 (1)	4898 (0)	4009 (3)	
Sc (1)	0.244 (1)	0.325 (0)	0.215 (3)	0.291 (0)	0.157 (5)	0.172 (5)	0.222 (2)	0.401 (0)	0.217 (2)	0.229 (1)	0.253 (1)	0.102 (5)	0.204 (3)	
V^a^ (0)	7.92 (2)	10.82 (0)	7.92 (2)	11.18 (0)	7.96 (2)	7.33 (2)	8.71 (0)	16.47 (0)	6.09 (2)	8.3 (1)	8.32 (1)	4.88 (3)		24 (0)
Cr^a^ (0)	2.97 (0)	4.76 (0)	2.97 (0)	4.96 (0)	2.78 (1)	3.62 (0)	3 (0)	6.49 (0)	2.72 (2)	2.79 (1)	2.89 (1)	1.64 (4)	2.93 (1)	50 (0)
Co^a^ (0)	0.99 (1)	1.51 (0)	0.99 (1)	1.56 (0)	1.03 (1)	1.19 (0)	1.09 (0)	2.29 (0)	0.84 (2)	0.9 (1)	0.98 (1)	0.53 (5)	0.97 (1)	3 (0)
Ni (2)	4.03 (1)	4.46 (1)	2.65 (4)	3.14 (2)	2.72 (2)	2.76 (2)	3.19 (2)	4.71 (1)	2.81 (2)	3.84 (1)	4.72 (1)	2.71 (2)	2.84 (2)	9 (0)
Cu (2)	4.69 (2)	8.03 (0)	3.17 (3)	5.79 (0)	3.51 (3)	4.4 (2)	4.25 (2)	10.71 (0)	3.85 (2)	5.1 (1)	5.25 (1)	2.52 (3)	2.89 (3)	8 (0)
Zn (1)	90.9 (1)	104.8 (1)	78.5 (2)	92.3 (1)	60.9 (5)	63.3 (4)	82.9 (2)	126.7 (0)	74.5 (2)	84.7 (1)	97.1 (1)	42.5 (5)	76.9 (2)	35 (9)
As^a^ (1)	3.12 (1)	5.11 (0)	2.64 (2)	4.62 (0)	2.62 (2)	2.6 (2)	2.91 (2)	5.23 (0)	2.13 (2)	3.01 (1)	3.44 (1)	2.03 (2)	2.21 (2)	3 (1)
Se (2)	0.37 (1)	0.31 (1)	0.1 (2)	0.14 (2)	0.11 (2)	0.11 (2)	0.14 (2)	0.21 (2)	0.11 (2)	0.28 (1)	0.49 (1)	0.09 (2)	0.13 (2)	
Rb (0)	1.69 (2)	2.23 (0)	1.69 (2)	2.3 (0)	1.35 (3)	1.51 (2)	1.74 (1)	3.35 (0)	1.45 (2)	1.73 (1)	1.76 (1)	1.78 (0)	1.58 (2)	47 (0)
Mo (2)	3.12 (2)	5.22 (0)	2.01 (2)	3.74 (0)	2.42 (2)	3.03 (2)	3.02 (2)	10.11 (0)	2.1 (2)	3.46 (1)	3.59 (1)	1.95 (2)		0.49 (14)
Ag (3)	0.309 (1)	0.308 (1)	0.113 (4)	0.136 (3)	0.130 (3)	0.138 (3)	0.155 (3)	0.238 (2)	0.204 (2)	0.294 (1)	0.394 (1)	0.119 (3)	0.122 (3)	
Cd (1)	1.699 (1)	2.558 (0)	1.176 (3)	2.044 (1)	1.191 (3)	1.144 (3)	1.395 (1)	6.943 (0)	1.260 (2)	1.371 (1)	2.032 (1)	0.677 (3)	1.292 (2)	0.180 (14)
Sb (1)	0.85 (1)	1.08 (1)	0.49 (2)	0.81 (1)	0.5 (2)	0.53 (2)	0.6 (1)	1.61 (0)	0.49 (2)	0.61 (1)	1.06 (1)	0.58 (1)	0.51 (2)	0.43 (3)
Ba^a^ (0)	28.8 (1)	31.7 (0)	28.8 (1)	32.3 (0)	28 (2)	28.6 (1)	30.7 (1)	41.7 (0)	23.6 (2)	28.5 (1)	29.9 (1)	23 (2)		312 (0)
Re^a^ (0)	0.457 (1)	0.999 (0)	0.457 (1)	1.052 (0)	0.466 (0)	0.6 (0)	0.579 (0)	1.603 (0)	0.366 (2)	0.4 (1)	0.437 (1)	0.397 (1)	0.4 (1)	
Tl (1)	0.175 (1)	0.274 (0)	0.117 (2)	0.222 (1)	0.119 (2)	0.14 (2)	0.146 (1)	0.446 (0)	0.084 (2)	0.147 (1)	0.215 (1)	0.078 (2)	0.144 (1)	0.12 (2)
Pb^a^ (0)	13.3 (2)	30.1 (0)	13.3 (2)	31.8 (0)	11.5 (2)	11.2 (2)	14.3 (1)	31 (0)	9.8 (2)	13.8 (1)	14.3 (1)	9.4 (2)	11.7 (2)	14 (1)
Bi^a^ (0)	0.244 (2)	0.361 (0)	0.244 (2)	0.374 (0)	0.244 (2)	0.259 (0)	0.289 (0)	0.624 (0)	0.173 (2)	0.249 (1)	0.251 (1)	0.164 (2)	0.205 (2)	
Th^a^ (0)	0.17 (0)	0.209 (0)	0.17 (0)	0.214 (0)	0.161 (2)	0.151 (2)	0.189 (0)	0.249 (0)	0.144 (2)	0.163 (1)	0.164 (1)	0.142 (2)	0.146 (2)	7 (0)
U (2)	1.22 (1)	2.38 (0)	0.59 (3)	1.48 (1)	0.75 (2)	1.51 (1)	0.92 (2)	7.88 (0)	0.66 (2)	1.04 (1)	1.55 (1)	0.62 (2)	0.66 (2)	1 (1)
**Rare earth elements—REEs**														
La (1)	3.41 (1)	4.65 (0)	2.61 (2)	3.95 (1)	2.58 (2)	2.89 (1)	3.18 (1)	6.14 (0)	2.33 (2)	2.87 (1)	3.9 (1)	2.78 (1)		17.6 (0)
Ce (1)	8.09 (1)	11.02 (0)	6.16 (2)	9.26 (1)	5.82 (2)	6.47 (2)	7 (2)	13.52 (0)	5.4 (2)	7.37 (1)	9.49 (1)	7.18 (1)	7.27 (1)	34.9 (0)
Pr (1)	0.8 (1)	1.134 (0)	0.661 (2)	0.995 (0)	0.679 (2)	0.687 (2)	0.798 (1)	1.578 (0)	0.554 (2)	0.784 (1)	0.903 (1)	0.53 (2)	0.754 (2)	3.7 (0)
Nd (1)	3.56 (1)	4.82 (1)	2.67 (2)	4.04 (1)	2.96 (1)	2.81 (2)	3.37 (1)	6.93 (0)	2.38 (2)	3.05 (1)	4.14 (1)	2.32 (2)	3.26 (1)	15.1 (0)
Sm (1)	0.741 (1)	1.064 (0)	0.567 (2)	0.898 (1)	0.571 (2)	0.573 (2)	0.658 (2)	1.356 (0)	0.454 (2)	0.718 (1)	0.883 (1)	0.426 (2)	0.655 (2)	2.9 (0)
Eu (2)	0.166 (2)	0.24 (0)	0.1 (2)	0.167 (2)	0.135 (2)	0.13 (2)	0.16 (2)	0.497 (0)	0.099 (2)	0.177 (1)	0.198 (1)	0.091 (2)	0.142 (2)	5.7 (0)
Gd (2)	1.165 (1)	1.361 (1)	0.61 (2)	0.881 (2)	0.78 (2)	0.714 (2)	0.856 (2)	1.658 (1)	0.636 (2)	0.972 (1)	1.451 (1)	0.603 (2)	1.044 (1)	2.75 (0)
Tb (2)	0.124 (1)	0.142 (1)	0.062 (2)	0.091 (2)	0.081 (2)	0.074 (2)	0.092 (2)	0.185 (1)	0.065 (2)	0.1 (1)	0.155 (1)	0.062 (2)	0.093 (2)	0.43 (0)
Dy (1)	0.581 (1)	0.881 (0)	0.399 (2)	0.718 (1)	0.413 (2)	0.466 (2)	0.477 (2)	1.155 (0)	0.32 (2)	0.499 (1)	0.71 (1)	0.302 (2)	0.551 (1)	2.5 (0)
Ho (2)	0.086 (2)	0.13 (0)	0.057 (2)	0.096 (2)	0.068 (2)	0.066 (2)	0.078 (2)	0.167 (0)	0.06 (2)	0.097 (1)	0.098 (1)	0.056 (2)	0.071 (2)	0.5 (0)
Er (2)	0.334 (1)	0.495 (1)	0.157 (2)	0.323 (1)	0.2 (2)	0.219 (2)	0.217 (2)	0.495 (1)	0.15 (2)	0.267 (1)	0.424 (1)	0.138 (2)	0.269 (1)	1.47 (0)
Tm (1)	0.035 (1)	0.078 (0)	0.022 (2)	0.065 (0)	0.024 (2)	0.025 (1)	0.028 (1)	0.086 (0)	0.02 (2)	0.026 (1)	0.042 (1)	0.025 (1)	0.031 (1)	0.22 (0)
Yb (0)	0.135 (0)	0.21 (0)	0.135 (0)	0.218 (0)	0.157 (0)	0.205 (0)	0.178 (0)	0.706 (0)	0.109 (2)	0.119 (1)	0.125 (1)	0.129 (0)		1.41 (0)
Lu (2)	0.037 (1)	0.055 (1)	0.017 (2)	0.035 (1)	0.024 (2)	0.023 (2)	0.026 (2)	0.071 (0)	0.016 (2)	0.03 (1)	0.047 (1)	0.014 (2)	0.029 (1)	0.22 (0)

^a^Normal distribution.

**Figure 2:**
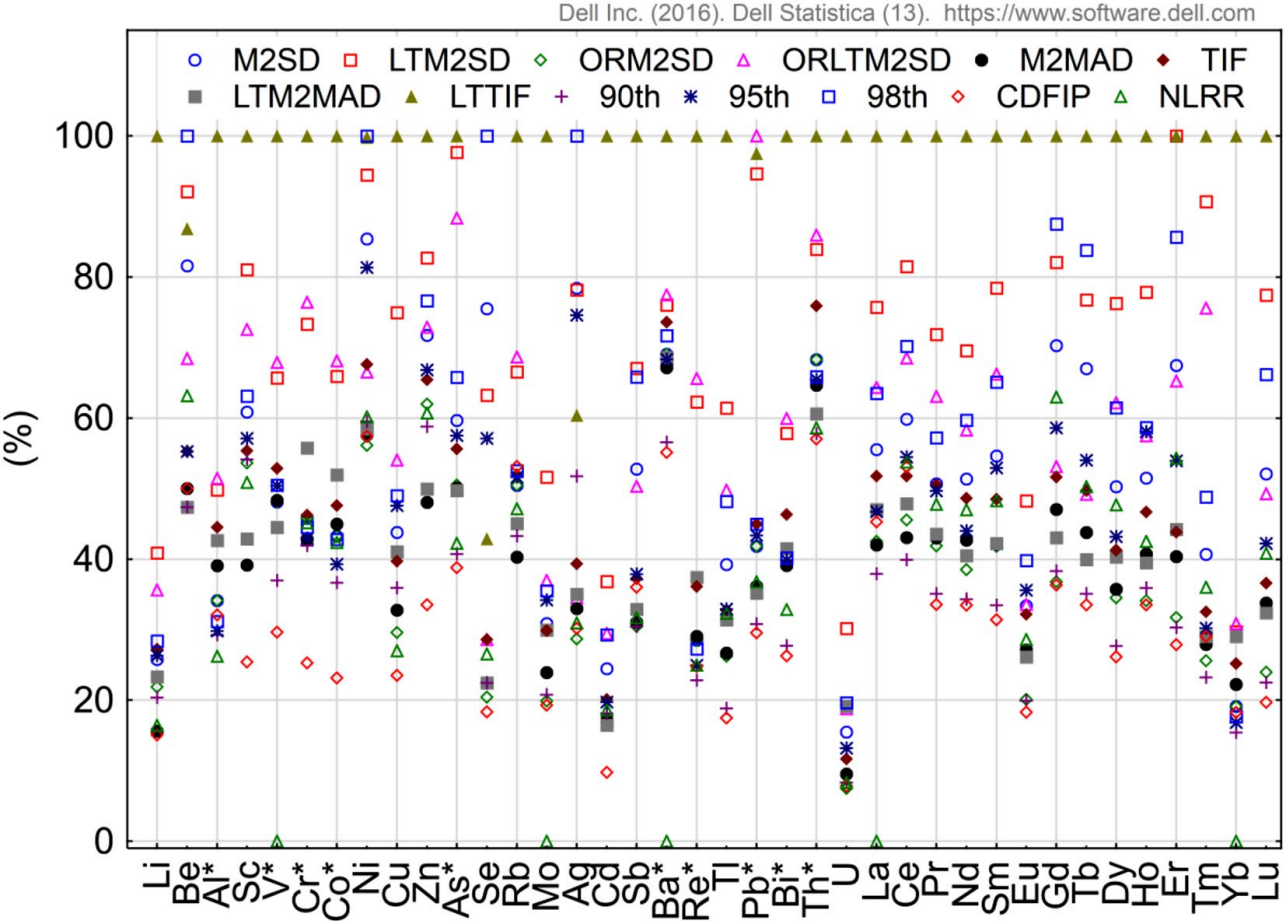
Variability in relative GBV values obtained by different methods (*elements having normal distribution). Figure generated in Statistica 13. (https://software.dell.com/products/statistica/).

The descriptions of methods are presented in Table 2. The EGA values were obtained from the European Geochemical Atlas developed by Salminen et al.^56^ for the BTNP location. In order to compare the results obtained with different methods, they were standardized in relation to the maximum value (Fig. 2).

The results showed that the differences between the minimum and maximum GBV values were for U 92.5% (the highest value) and for Th 43.0% (the lowest value). Average differences between the minimum and maximum GBV values within TEs and REEs were about 71%. The highest GBV values were obtained using LTTIF method, while the lowest values were obtained using CDFIP method. Using the LTTIF method, very high GBV values were obtained. For 31 elements, their concentrations in bottom sediment were lower than GBV. For Be, Ni, Gd, Tb and Er only 1 value exceeded GBV value and for Se and Ag only 2 values. Also high GBV values were obtained using LTM2SD method. For 27 elements the maximum concentrations did not exceed GBV value and for 11 elements only one value was higher than GBV. Using LTM2SD methods, GBV values were on average 30% higher than those of the 98th percentile. The lowest GBV values were obtained using the CDFIP method. In this case, GBV values were on average about 10% lower than the value corresponding to the 90th percentile. However, for Sc, Cr, Co, Cu, Zn, Ag and Cd the GBV values obtained by means of the CDFIP method were over 30% lower than those corresponding to the 90th percentile. In case of small research objects such as BTNP it is very important to thoroughly analyse the whole set of measurement data. From the methodological point of view the CDFIP method is the most suitable one, in which the inflexion point is detected. This point enables indication within a series of data of those potentially related to anthropogenic sources. The application of a method based on CDF enables detection of such observations, which differs from the others. The analysis showed that among TEs such elements were detected in samples taken from the western part of Ostrowite Lake (16 times), Krzywce Wielkie (ten times), the eastern part of Ostrowite Lake (seven times), Głuche (six times), Krzywce Małe (four times), Bełczak (three times), Płęsno, Gacno Wielkie and Główka (two times) and Jeleń, Zielone, skrzynka, Gacno Małe, Mielnica i Nierybno (one times). There were no outliers when analyzing CDF for TEs in the case of Olbrachta, Kocioł, Kociołek, Kacze Oko and Rybie Oko lakes. In the case of REEs outlier values were found only in the western and eastern part of Ostrowite Lake 13 and 10 times, respectively. Taking into account individual elements, the greatest number of outliers was found in Sc, Co and Zn (five times), Li and Cr (four times) and V, Cu, Ag and Cd (three times). However, in the case of Al and Rb, outliers were not detected. Among REEs there were usually two outliers (ten times). Only in the case of Yb no outliers were observed. The standard approach to analysis using M2SD, M2MAD, LTM2MAD, TIF and LTTIF methods does not allow one to detect values that could potentially be associated with anthropogenic sources. Detailed statistical analysis allows one, on the one hand, to remove outliers (ORM2SD), transform variables to a normal distribution (LTM2SD) or perform the above operations together (ORLTM2SD), but still these methods do not allow one to detect suspicious observations. In the case of the NLRR method, which was to normalize TEs against V and REEs against La, the observations outside the 95% confidence interval were removed. These observations are considered to be atypical. When NLRR was used, significantly more atypical values were identified among TEs. For western and eastern parts of Ostrowite Lake, Jeleń, Płęsno, Krzywce Małe, Nierybno, Główka, Bełczak, Głuche and Olbrachta from 10 to 18 atypical parameters were identified out of 24 examined. There were no lakes without atypical values among TEs. In the case of REEs, 5–10 atypical values were detected in lakes Ostrowite, Krzywce Wielkie, Gacno Małe, Krzywce Małe, Bełczak, Głuche and Olbrachta. Only in the case of Lake Gacno Wielkie were no atypical values observed among REEs. Taking into account individual elements, the most atypical values were found among Al (12), Pb, Th and Gd (11) and Sc, Re, Bi and U (10). The least atypical values were identified within Pr (3) and Cr and Nd (4). The results show that it is a method that does not always exclude outliers from the data set. These are rather atypical values resulting from the different geological structure of geochemical processes rather than from the influence of anthropogenic sources. In addition, calculations have shown that there is no relationship between V and Mo and Ba, and La and Yb. In this respect, another element for standardisation should be identified, which may have an impact on the homogeneity of GBV results. The results show that the calculation of the GBV value requires an individual approach for each element. Taking into account the above results, it was considered that the choice of the CDFIP method is the most appropriate one. On the one hand, lakes where bottom sediments may have been contaminated have been identified. On the other hand, data sets within individual elements have been reduced by up to five values associated with anthropogenic sources. GBV results calculated with the CDFIP method were used to determine the contamination of sediments by means of geochemical indices I_geo_, EF and PLI.

**Figure 3:**
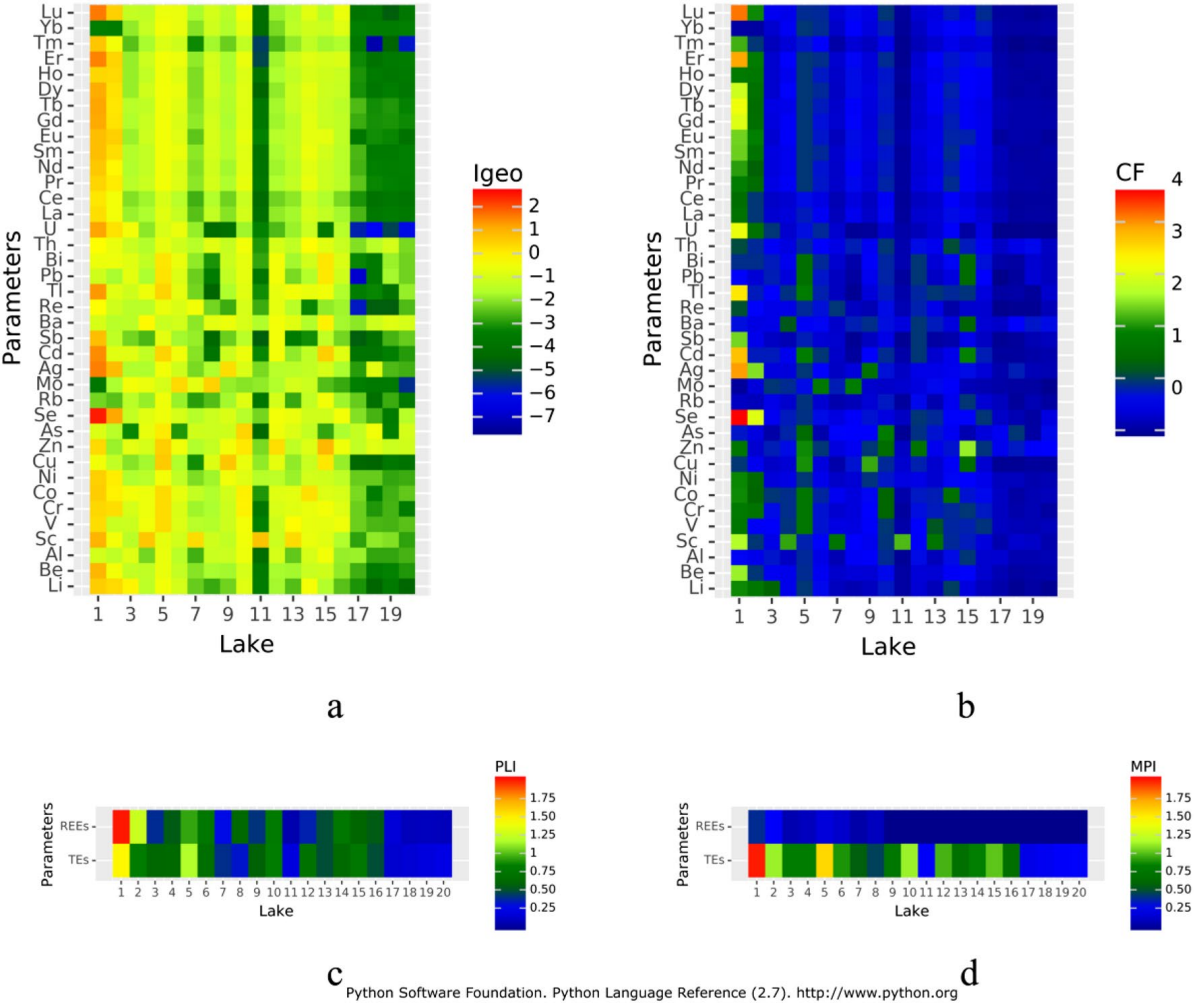
Contamination of lake bottom sediments calculated on the basis of Igeo (**a**), CF (**b**), PLI (**c**) and MPI (**d**) indices. Figure generated in Python Language Reference 2.7 (http://www.python.org).

Results obtained with the I_geo_ index indicate that in most lakes the bottom sediments were unpolluted (Fig. 3a). In lakes Jeleń (no. 3), Olbrachta (no. 16), Kocioł (no. 17), Kociołek (no. 18) and Kacze Oko (no. 19) and Rybie Oko (no. 20) Igeo values for all elements were lower than 0, which indicates that they were not contaminated. For most of the other lakes, single values of the I_geo_ index were higher (from 0 to 1), with the exceptions of Ostrowite (no. 1 and 2) and Krzywce Wielkie Lake (no. 5). In the eastern part of Ostrowite Lake (2) and Lake Krzywce Wielkie (2) the values of the I_geo_ index from 0 to 1 were 13 times and nine times, respectively. The most polluted were the bottom sediments of the western part of Ostrowite Lake (no. 1), 19 values of the I_geo_ index indicated their poor pollution (0 < I_geo_ ≤ 1), seven values moderate pollution (1 < I_geo_ ≤ 2) and in one case more than moderate pollution (I_geo_ > 2). Taking into account particular elements, moderate pollution was found for Ag, Cd, U, Tb, Er and Lu. However, in the case of Se, pollution exceeded the level of moderate pollution one time. The results of the CF index in most cases corresponded to the results of the I_geo_ index (Fig. 3b). However, the use of the CF index indicates slightly higher contamination of bottom sediments. The results showed that the most polluted were the sediments of the western part of Ostrowite Lake (1), and among 38 analysed elements as many as in 22 cases CF values indicated moderate pollution (1 < CF ≤ 3). Moreover, in seven cases the pollution was considerable (3 < CF ≤ 6) and in one case there was very high contamination (CF > 6). In the case of other lakes the CF index values were lower than considerably polluted and very highly polluted. For most lakes there were single elements, which indicated moderate contamination. A higher number of elements indicated moderate contamination in the case of the eastern part of lakes Ostrowite (no. 2)—22 elements, Krzywce Wielkie (no. 5)—23 elements, Głuche (no. 15)—nine elements and Krzywce Małe (no. 10) and Nierybno (no. 12)—seven elements. Taking into account individual elements, the values of CF index above 1 were most often noted in the cases of Li and Cr (five times) and Sc, Co and Zn (six times). In the case of Ag, Cd, Tl, U, Tb, Er and Lu, one value each was found to show considerable pollution and in the case of Se one value was found to show very highly pollution. PLI index analysis showed that for TEs values ranged from 0.17 to 1.45 and for REEs from 0.09 to 1.96. PLI values above 1, which indicates sediment contamination for TEs, were found for the western part of Ostrowite Lake (no. 1) and Krzywce Wielkie Lake (no. 5), whereas for REEs values for both parts of Ostrowite Lake (nos. 1 and 2) were found (Fig. 3c). The MPI values ranged from 0.23 to 1.95 and from 0 to 0.413 for TEs and REEs respectively. In the case of TEs, the most polluted were bottom sediments of the western part of Ostrowite Lake (no. 1) and Krzywce Wielkie Lake (no. 5). Moreover, MPI values higher than 1 were recorded for the eastern part of Ostrowite Lake (2), Krzywce Małe Lake (10) and Głuche Lake (no. 15) (Fig. 3d). In the case of REEs, both parts of Ostrowite Lake (nos. 1 and 2) were the most polluted.

**Figure 4:**
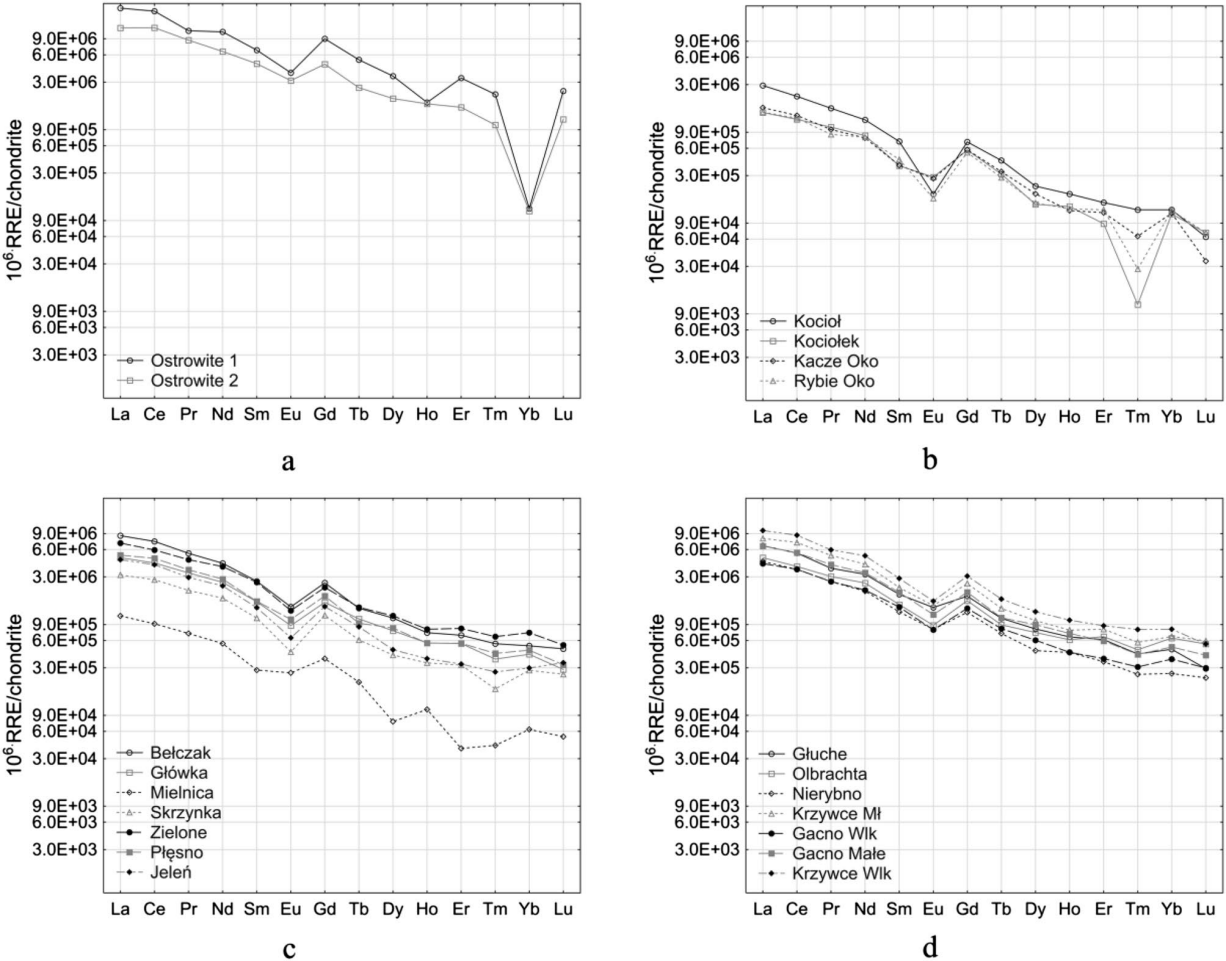
Standardized REE contents according to chondrite. Figure generated in Statistica 13. (https://software.dell.com/products/statistica/).

The analysis of REEs concentration in bottom sediments of the BTNP lakes was started with the development of standardized graphs with respect to their concentrations in chondrite (Fig. 4). In terms of REEs the lakes were divided into three groups: (1) Ostrowite Lake—western and eastern part (Fig. 4a), (2) Kacze Oko, Rybie Oko, Kocioł and Kociołek lakes (Fig. 4b) and (3) other lakes (Fig. 4c,d) excluding Mielnica Lake. In the case of this reservoir, the standardized REE graph differed from all other lakes. Visual analysis of the graphs showed the occurrence of a negative Eu anomaly and a positive Gd anomaly in most lakes. Moreover, in Ostrowite Lake and additionally in Kocioł, Kacze Oko and Rybie Oko a negative anomaly of Yb and Tm was observed respectively. The formulas presented by Benabdelkader et al.^65^ were used to confirm the above observations. It was assumed that the computations of Eu, Gd, Yb and Tm anomalies will have values above > 1.1 or below < 0.9, which indicates the occurrence of positive and negative anomalies, respectively. A value of 1 means that the element was not fractionated relative to the crustal composition^62^. The calculations confirmed the occurrence of negative Eu anomalies, except for Mielnica Lake. The values of Eu anomalies ranged from 0.30 to 0.84. A similar negative Eu anomaly was found in the bottom sediments of Lake Wadąg^32^. The lowest values were recorded for Kocioł and Rybie Oko lakes 0.30 and 0.42, respectively. A positive Gd anomaly was found for all lakes in the range from 1.28 to 1.71. Yb analysis showed a very strong negative anomaly for Ostrowite Lake (0.05 and 0.10) and a very strong positive anomaly for Kociołek, Kacze Oko and Rybie Oko lakes (from 2.34 to 2.76). In the case of Kociołek and Rybie Oko lakes there were negative Tm anomalies of 0.14 and 0.26, respectively.

**Figure 5:**
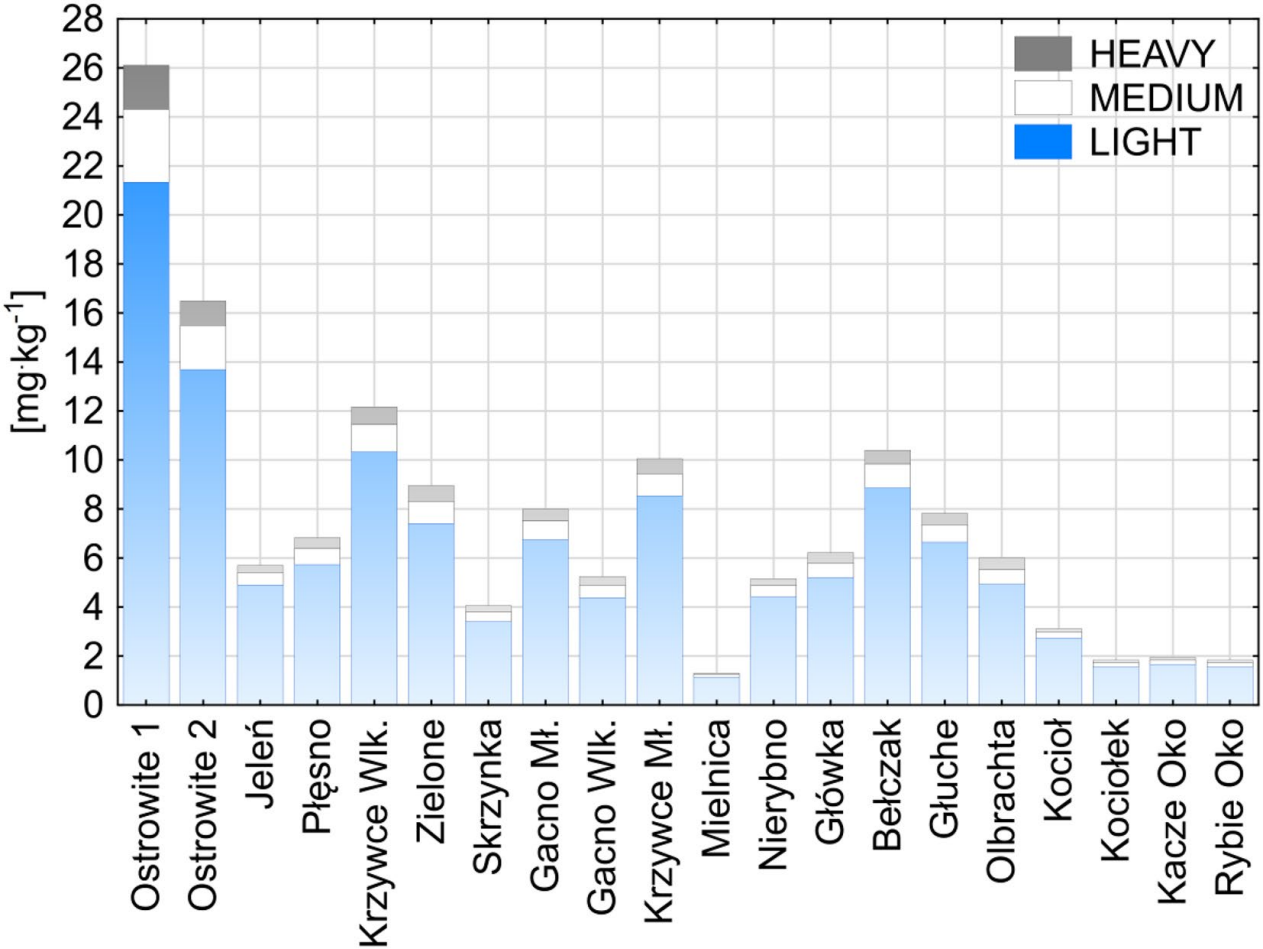
Cumulative REE contents in lake bottom sediments divided into light, medium and heavy fractions. Figure generated in Statistica 13. (https://software.dell.com/products/statistica/).

REE analysis in particular groups showed that LREE dominated over MREE and HREE in bottom sediments (Fig. 5). This is confirmed by earlier studies by Sojka et al.^32^, obtained for Lake Wadąg located in north-eastern Poland. Benabdelkader et al.^65^ demonstrated that reservoirs play an important role in REE retention (LREE) and to delay the release of MREE and HREE downstream. There was slightly more MREE in the individual lakes than HREE. Only in the case of Kociołek, Kacze Oko and Rybie Oko lakes were the MREE and HREE contents at a similar level. The (La/Yb)_N_ ratio normalized in relation to chondrite in the bottom sediments of the BTNP lakes ranged from 7.7 to 160.4. The lowest (La/Yb)_N_ ratio was observed in Lake Olbrachta (7.7) and the highest in the western and eastern parts of Ostrowite Lake (160.4 and 103.7, respectively). In the other lakes the values (La/Yb)_N_ ranged from 9.6 to 23.4. The obtained values were generally lower than that obtained by Sultan and Shazili^71^ which was about 20.6.

**Figure 6:**
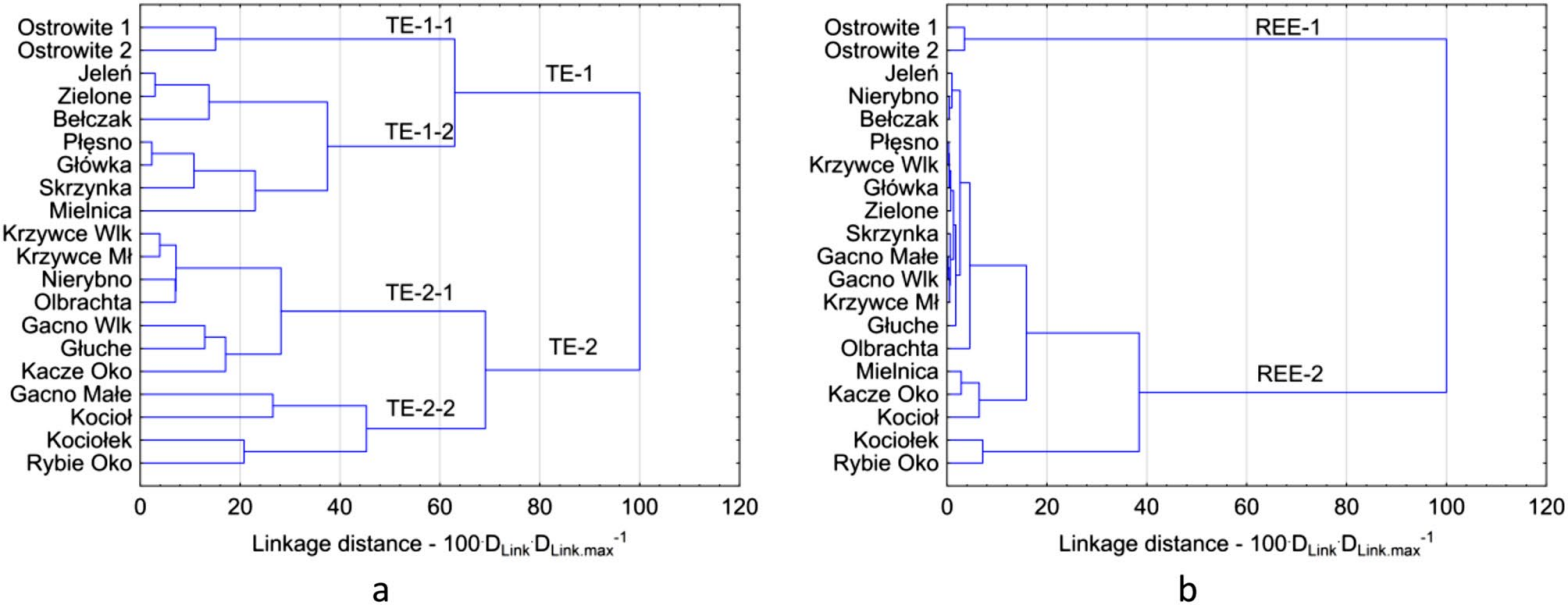
CA analysis results for TEs (**a**) and REEs (**b**). Figure generated in Statistica 13. (https://software.dell.com/products/statistica/).

The CA analysis for TEs enabled the division of lakes into two main groups: TE-1 and TE-2 (Fig. 6a). Within each of the groups, two subgroups have been distinguished. The TE-1–1 subgroup includes western and eastern part of Ostrowite Lake with the highest concentrations of Li, Be, Sc, V, Cr, Co, Ni, Se, Ag, Cd, Sb, Re, Tl, Bi, Th and U. The lowest TE concentrations were found in the TE-2–2 subgroup, which included four lakes: Gacno Małe, Kocioł, Kociołek and Rybie Oko. The TE-1–2 subgroup includes seven lakes, connected by the Struga Siedmiu Jezior. The concentrations in the TE-1–2 subgroup were slightly lower than in the TE-2–1 subgroup, except for Li, Mo, U and Sc. It should be noted that the highest differences in TE concentration were found in the TE-2 group, which included 11 lakes. However, this is due to the lack of direct connection between the lakes and the various geological structure. In the case of REE concentrations in bottom sediments, lakes were divided into two groups: REE-1 and REE-2 (Fig. 6b). REE-1 includes both parts of Ostrowite Lake, in which REEs concentrations were at the highest level. However, within the REE-2 group REEs concentrations were lower, but there was a relatively large variation between individual lakes. In general, the lowest REEs concentrations were found in Mielnica, Kociołek, Kociołek, Kacze Oko and Rybie Oko lakes.

**Figure 7:**
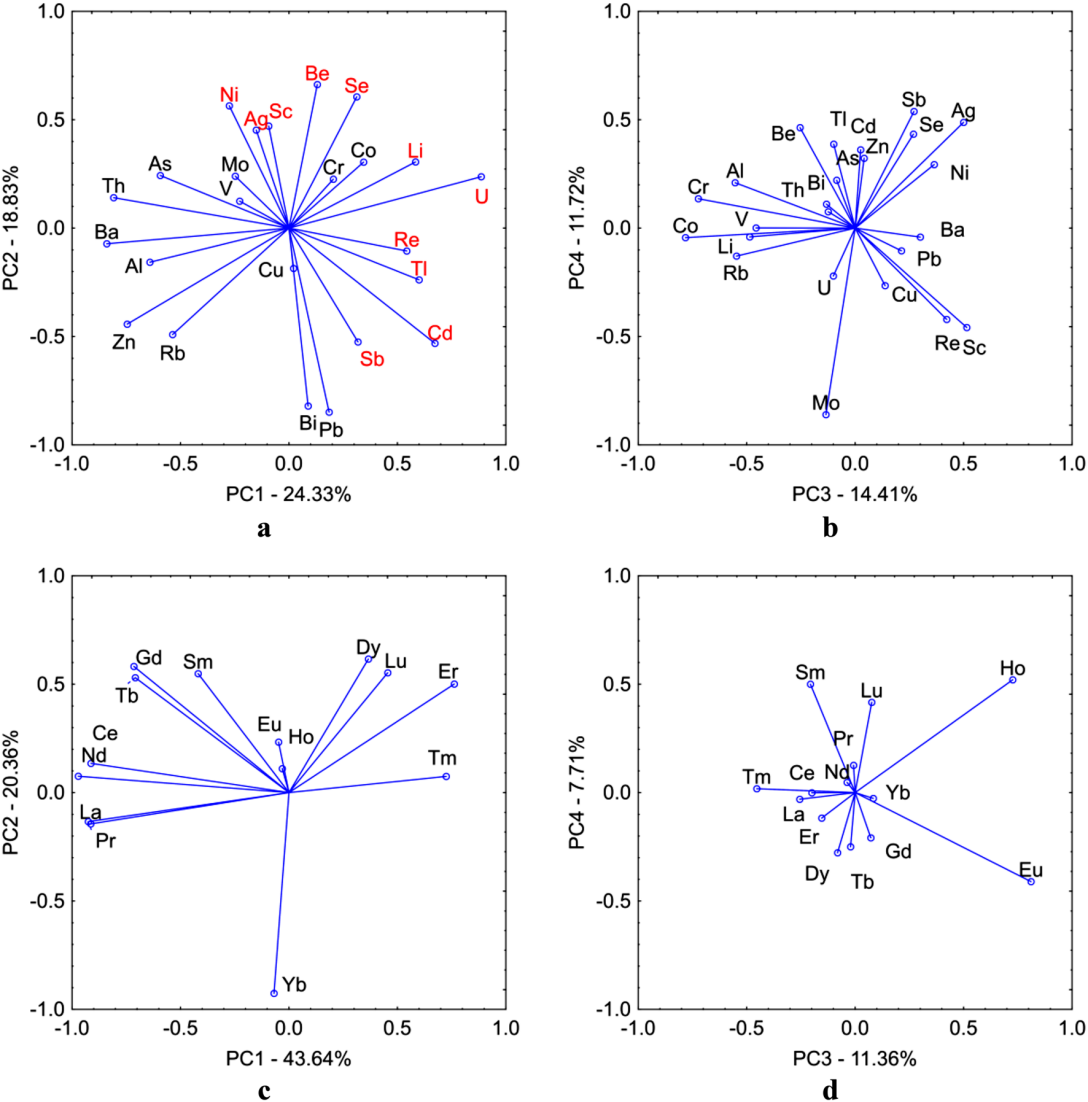
The results of PCA analysis for TEs PC1 vs. PC2 (**a**), PC3 vs. PC4 (**b**) and for REEs PC1 vs. PC2 (**c**), PC3 vs. PC4 (**d**). Figure generated in Canoco 5.0 (http://www.microcomputerpower.com).

PCA analysis in the case of TEs allowed us to distinguish six significant components, whose eigenvalues were higher than 1. The components explain 83.10% of the internal data structure (Table 5). With the first PC1 component, Zn, Ba and Th concentrations were strongly negatively correlated and U concentrations were strongly positive correlated. Moreover, with PC1 the concentrations of Al, As and Rb and the concentration of Li, Cd, Re and Tl were respectively moderate negatively and positively correlated. A correlation with PC2 was noted with eight elements. For Pb and Bi strong negative correlation, Cd and Sb moderate negative correlation and for Be, Ni and Se moderate positive correlation. Al, Sc, Cr, Co, Rb and Ag (PC3), Mo and Sb (PC4), V and As (PC5) and Cu (PC6) were correlated with subsequent components respectively. The projection of the elements against the background of the four components is shown in Fig. 7a,b. The concentrations of Li, Ni, Sc, Se, Be, Se, Ag, Re, Tl, Cd, Sb and U may be related to the impact of anthropogenic point sources found mainly in the Ostrowite Lake. However, Cu and Mo concentrations are associated with point sources of pollution occurring in other lakes. The other TEs comes mainly from geogenic sources. This is confirmed by the distribution of concentrations of these elements (generally normal) and the absence of outliers.

**Table 5:** Principal component loadings extracted from the geochemical centered log-ratio transformed lake sediments data.

Parameters	Components	Elements association
Trace elements—TEs	PC1 (24.33%)	Li–Cd–Re–Tl–U (+ vely loaded)
		Al–Zn–As–Rb–Ba–Th (− vely loaded)
	PC2 (18.83%)	Be–Ni–Se (+ vely loaded)
		Cd–Sb–Pb–Bi (− vely loaded)
	PC3 (14.41%)	Sc–Ag (+ vely loaded)
		Al–Cr–Co–Rb (− vely loaded)
	PC4 (11.72%)	Sb (+ vely loaded)
		Mo (− vely loaded)
	PC5 (8.60%)	V–As (− vely loaded)
	PC6 (5.22%)	Cu (− vely loaded)
Rare earth elements—REEs	PC1 (43.64%)	Er–Tm (+ vely loaded)
		La–Ce–Pr–Nd–Gd–Tb (− vely loaded)
	PC2 (20.36%)	Sm–Gd–Tb–Dy–Lu (+ vely loaded)
		Yb (− vely loaded)
	PC3 (11.36%)	Eu–Ho (+ vely loaded)
	PC4 (7.71%)	Sm–Ho (+ vely loaded)

The PCA analysis of REEs allowed us to distinguish four main components which explain 83.08% of the data structure (Table 5). There were nine elements correlated with PC1 and six elements correlated with PC2. Whereas Eu, Ho and Sm were correlated with PC3 and PC4. The projection of the elements against the background of the four components is shown in Fig. 7c,d. Analysis of REEs against the background of PC1 and PC2 components allows for their division into LREEs and MREEs and HREEs. The exception is Yb and Eu and Ho. For REEs, their distribution in bottom sediments is mainly related to natural geochemical processes, which is confirmed by the concentrations and strong inter-group correlations. The only exception is Lake Ostrowite, where there were outlier values, which indicate their anthropogenic origin. The concentrations of Yb in bottom sediments show considerably different spatial variation from other REEs. There were no outliers within the Yb concentrations. However, both negative and positive anomalies of Yb concentrations were recorded in lakes. In the case of Eu, almost all lakes negative anomalies were observed.

In order to expose the elements best describing the state of contamination of bottom sediments, multiple regression analysis was carried out. Relationships between individual TEs or REEs (independent variables) and values of PLI and MPI indices were investigated. For TEs the calculations showed that for both PLI and MPI the best model with 12 elements—Cr, Co, Cu, Ag, Cd, Zn, Bi, Re, Ba, Al and Rb—was used. The models were statistically significant at 0.05 and the determination coefficient was 0.99. In the case of REEs the results showed that the model for PLI was based on seven elements (Nd, Gd, Yb, Lu, Eu, Dy and Ce) and in the case of MPI only one element (Dy). The determination coefficients for the models for PLI and MPI were 0.76 and 0.99, respectively.

## Discussion

The studies indicate that among TEs and REE there were elements that were characterized by various variability, types of distributions and occurrence of outliers. The obtained results are somewhat unexpected as the studied lakes are located in a small area and were formed as a result of glacial processes of the last Central European glaciation. The surveyed area is located completely in the Struga Siedmiu Jezior catchment, which eliminates the inflow of pollutants from the external area. Moreover, the BTNP area is characterised by relatively stable forest land use in the last 120 years. Bottom sediment samples have also been taken from the deepest locations within each lake, away from the shore line. Most often higher concentrations are recorded in the littoral zone than in the profundal zone^32,72^. The studies presented in this paper should be considered as very valuable and may serve as a reference point for further geochemical studies in this area. Similar conclusions were drawn from the studies (Raut et al. 2017)^25^ for shallow to medium lakes in high-altitude mountains, which may be important archives for studying various types of pollutants. An essential stage of geochemical analysis is the determination of the GBV value, which is used to assess the contamination of bottom sediments. Gałuszka and Migaszewski^14^ indicated that only reliable GBVs enable one to establish objective environmental quality criteria. First of all, it is important to use GBV values calculated for a given region to obtain more reliable results^19,50,73^. Secondly, the choice of the method of estimating the GBV value itself is important. In this paper, 13 methods of GBV calculation were tested. The analysis showed that the uncertainty related to the choice of method for GBV determination, expressed as the difference between the minimum and the maximum value, is at the level of 70%. However, in extreme cases it can reach as high as 90%. Moreover, statistical methods, although to varying degrees they enable one to detect outliers, do not allow observations to be divided into groups which are connected with natural and anthropogenic processes. The results obtained by Reimann and Garrett^24^ suggest that in the case of small data sets or irregular statistical distribution, methods based on median value should be used. Among the statistical methods Reimann et al.^21^ recommends the mMAD and TIF methods with log-transformed data. The method that reliably allowed detection of observations different from the general population was the one based on the CDF on which the inflexion point is detected^74^. The higher inflexion point represents the lower limit of abnormal concentrations^51^. Similarly, Reimann and Caritat^50^ recommend the method of identifying breaks in cumulative probability distributions for determining the GBV value, as well as the application of the Tukey inner fence and the 98th percentile. Zhou et al.^12^ using the cumulative frequency method and the normalization method, obtained similar results. In this study, the application of the method of normalization (NLRR) to V and La caused that many so-called abnormal values were removed from the TE and REE set. The results obtained by NLRR and CDFIP methods differed even by more than 50% in the case of some elements. The calculated GBV values are used later to determine the degree of contamination of sediments by means of geochemical indices, which are calculated for each element (single indices) or for the whole set of TEs and REEs (integrated indices). GBV values enable one to assess possible anomalies and to provide information on the diffusion and dispersion patterns of pollutants inside the monitored area^53^. In addition, they may be useful for identifying the pollution source^15^. The results of Igeo, EF, PLI and MPI indices indicate that TE and REE concentrations in the bottom sediments of the BTNP lakes result mainly from natural geochemical processes. In individual cases, higher concentrations may indicate the occurrence of anthropogenic sources. Similar results were obtained by Raut et al.^25^, which indicated that only in the case of a few elements was the influence of anthropogenic factors, mainly long-range transport of atmospheric pollutants, observed. Strong correlation between REEs with the exception of Yb indicates the same source of origin of this group of elements. Slightly different results were obtained by Wang et al.^8^, who also observed strong inter-group correlations with the exception of Ce, Eu and Gd. The difference in results may be related to different degrees of anthropogenic source interactions.

Another important element associated with geochemical analyses of bottom sediments of very large data sets is the division of samples into groups characterized by similar concentrations and identification of sources of elements in bottom sediments. Sahoo et al.^68^ stated that the types of statistical processing and data manipulation are critical for understanding the role of various factors in the final geochemistry of the lake sediments. Multidimensional statistical methods are most frequently used for this purpose, among which cluster analysis and principal component analysis dominate^9,27–29^. Aitchison^66^, Filzmoser et al.^67^, Sahoo et al.^68^ indicates that data transformation should be carried out before statistical analysis. The clr transformation destroys the closure effect and ‘opens’ or ‘un-constrains’ the data to reveal inherent patterns in the data structure that can provide straight forward interpretation. In addition, the selection of representative elements to characterise the contamination of bottom sediments is important to simplify the analysis of large data sets. As this study has shown, it is possible to apply multiple regression models, which allow one to identify relationships between the TEs and REEs and the geochemical indices of PLI and MPI.

## Conclusion

The analysis of the study showed that:

- the variation of TEs and REEs in the bottom sediments of the lakes located in the BTNP is mainly due to the specific geological structure of this area, which determines the geochemical processes,
- anthropogenic sources only for Ostrowite and Krzywce Wielkie lakes had an impact on concentrations of selected TEs and REEs,
- the calculation of the geochemical background value by different methods has an uncertainty of approximately 71% on average with variation of 43.0–92.5%,
- the most reliable method to determine the geochemical background value and also to detect atypical values is the method based on CDF and the inflexion point,
- the most effective elements for characterizing the pollution of bottom sediments of lakes in the BTNP among TEs are: Cr, Co, Cu, Ag, Cd, Zn, Bi, Re, Ba, Al and Rb,
- the most effective elements for characterizing the pollution of bottom sediments of lakes in the BTNP among the REEs are: Nd, Gd, Yb, Lu, Eu, Dy and Ce,
- the lakes in the BTNP can be divided into four and two subgroups with similar TE and REE contents, respectively.

## Supplementary Information

Supplementary Table S1.
