# Supplementary material for: Causes of variations of trace and rare earth elements concentration in lakes bottom sediments in the Bory Tucholskie National Park, Poland
Source: Sci Rep. 2021 Jan 8;11:244. doi: 10.1038/s41598-020-80137-z (PMC7794470; doi:10.1038/s41598-020-80137-z)
Supplement: Supplementary file 1 — Supplementary Table S1. [file 41598_2020_80137_MOESM1_ESM.docx]

Supplementary Information

**Causes of variations of trace and rare earth elements concentration in lakes bottom sediments in the Bory Tucholskie National Park, Poland**

Mariusz Sojka^a^, Adam Choiński^b^, Mariusz Ptak^b^, Marcin Siepak^b*^

^a^Faculty of Environmental Engineering and Spatial Management, Poznań University of Life Sciences, Piątkowska 94E, 60-649 Poznań, Poland

^b^Faculty of Geographical and Geological Sciences, Adam Mickiewicz University, Krygowskiego 10, 61-680 Poznań, Poland

*Corresponding author: Marcin Siepak, Department of Hydrogeology and Water Protection, Institute of Geology, Faculty of Geographical and Geological Sciences, Adam Mickiewicz University, 10 Bogumiła Krygowskiego Street, 61-680 Poznań, Poland, (marcin.siepak@amu.edu.pl)

**Table S1.** Comparison of certified mass concentration, determination value and recovery

| Element | Certified value [mg·kg^-1^] | Determination value [mg·kg^-1^] | Recovery (%) |
| --- | --- | --- | --- |
|  | CRM | ICP-QQQ |  |
|  | LGC 6187 |  |  |
| As | 24.0 ± 3.2 | 23.5 ± 0.9 | 97.9 |
| Cd | 2.7 ± 0.3 | 2.8 ± 0.2 | 103.7 |
| Cr | 84.0 ± 9.4 | 83.1 ± 3.1 | 98.9 |
| Cu | 83.6 ± 4.1 | 83.8 ± 1.1 | 100.2 |
| Pb | 77.2 ± 4.5 | 76.5 ± 1.8 | 99.1 |
| Ni | 34.7 ± 1.7 | 34.1 ± 0.7 | 98.3 |
| Se | 1.2 ± 0.2 | 1.1 ± 0.1 | 91.7 |
| V | 38.3 ± 6.5 | 38.5 ± 2.0 | 100.5 |
| Zn | 439 ± 26 | 436 ± 5.9 | 99.3 |
